# Resilience Protected against Suicidal Behavior for Men But Not Women in a Community Sample of Older Adults in Korea

**DOI:** 10.3389/fpsyg.2017.00401

**Published:** 2017-03-15

**Authors:** Sungeun You, Moran Park

**Affiliations:** Department of Psychology, Chungbuk National UniversityCheongju, South Korea

**Keywords:** suicide, suicidal behavior, older adults, resilience, gender difference

## Abstract

Suicide prevention efforts in reducing risk factors have been found to be more beneficial to older women than men, suggesting potential gender differences in effective prevention. The study aimed to examine gender difference in resilience for suicidal behavior in a community sample of older adults in Korea. A community-based survey was conducted to investigate resilience and risk factors of suicidal behavior using the Suicidal Behaviors Questionnaire-Revised, Connor-Davidson Resilience Scale, Center for Epidemiologic Studies Depression Scale (CES-D), as well as questions regarding physical illness and depression history. After accounting for well-known risk factors, resilience was inversely associated with suicidal behavior, but this protective role of resilience was applicable to men only. The findings of this study indicated gender difference in resilience against suicidal behavior in the elderly population. Gender-specific preventive intervention strategies need to be developed for community-based suicide prevention for older adults.

## Introduction

Suicide rates in older adults have increased at a faster pace than any other age group over the past decades in Korea (World Health Organization [WHO], 2014). National suicide rates among Korean older adults aged 65 years and older were considerably high, with 95.2 in men and 32.1 in women (Statistics Korea, 2016). Considering that it is a rapidly growing population, not only in Korea but also the world (World Health Organization [WHO], 2012), it is critical to improve our understanding of suicide among the elderly. While incidence rates of suicidal ideation and attempt decreases with age, suicide rate increases as one get older (De Leo et al., 2001; Centers for Disease Control, and Prevention, 2015), suggesting higher likelihood of suicide death among older adults who have thought or attempted suicide compared to other age groups. This tendency seemed stronger for older men than older women (Rubenowitz et al., 2001; Paraschakis et al., 2012), although no consistent gender difference in prevalence of suicidal ideation was reported for older adults (Almeida et al., 2012; Park and Lee, 2015; Kim et al., 2016).

Despite this well-known gender difference in suicidal behavior in the elderly population, our knowledge regarding how older men and women differ in terms of risk and protective factors for suicidal behavior is limited. The most well-known risk factors for elderly suicide are depression and social isolation (Conwell et al., 2002, 2011), and preexisting elderly suicide prevention programs mostly rely on reducing these risk factors (Oyama et al., 2005; Unützer et al., 2006; Alexopoulos et al., 2009). Using randomized controlled trials, both IMPACT (Improving Mood-Promoting Access to Collaborative Treatment, Unützer et al., 2006) and PROSPECT (Prevention of Suicide in Primary Care Elderly: Collaborative Trial, Alexopoulos et al., 2009) programs resulted in a significant reduction in depressive symptoms and suicide risk for depressed older patients. Community-based intervention programs (Oyama et al., 2005, 2008), including depression screening, health education, and group activities were effective in reducing suicide risk for older adults. Interestingly, these empirically supported suicide prevention programs were more beneficial to older women than older men (Oyama et al., 2005, 2008; Drapeau et al., 2009). Moreover, older men and women differed in participation rates and preferences for intervention programs (Drapeau et al., 2009). In fact, older women were more likely to participate in intervention programs utilizing social groups, counseling, and other mental health services while older men were more inclined to engage in action-oriented or problem-solving oriented intervention programs (Oyama et al., 2008; Drapeau et al., 2009; Lapierre et al., 2011).

Gender differences in the effects or participation rates of suicide prevention programs among older adults suggest the need for developing differential strategies to suicide prevention for each gender. More understanding of gender-specific risk and protective factors would enable us to develop gender-specific intervention programs. As evidenced by psychological autopsy studies, depression is one of the strongest risk factors for elderly suicide (Beautrais, 2002; Conwell et al., 2002, 2011) while associations of other psychiatric disorders with suicide in older adults varied across samples and cultural backgrounds (Conwell et al., 2011). In addition, physical illnesses in old ages increased the risk for suicide (Juurlink et al., 2004; Conwell et al., 2010; Erlangsen et al., 2015). However, considering that late-life depression and physical illnesses are common risk factors for morbidity and mortality in this population (Schulz et al., 2002).

Current literature has rarely examined the role of protective factors for elderly suicide. Moreover, gender differences in such protective factors are largely unknown. A few studies examined the association between low resilience and suicidal behavior, although findings have been rather mixed. In a 3-year longitudinal study, resilience had a protective effect for suicidal ideation among veterans even after controlling for baseline suicidal ideation (Youssef et al., 2013). Similarly, low resilience was linked to suicide attempt in substance-dependent outpatients or prisoners (Roy et al., 2007, 2011). On the contrary, Liu et al. (2014, 2016) reported no relationship between resilience and suicidal ideation when several psychological covariates were controlled.

Resilience is a broad term denoting one’s ability to cope with life stress or adversity (Connor and Davidson, 2003; Windle et al., 2011). Based on several personality theories (Kobasa, 1979; Rutter, 1985; Lyons, 1991), Connor and Davidson (2003) developed a measure to quantify one’s resilience, which they called the Connor-Davidson Resilience Scale (CD-RISC). Connor and Davidson (2003) viewed resilience as a modifiable psychological construct that could be enhanced by clinical intervention. Indeed, a number of studies have supported successful modifications of resilience following various forms of intervention (Davidson et al., 2005; Steinhardt and Dolbier, 2008; Loprinzi et al., 2011; Stephens, 2013).

The study aimed to examine gender difference in the protective role of resilience against suicidal behavior among the elderly, especially after controlling for common risk factors associated with elderly suicide, such as sociodemographic risk factors, depression, and physical illness. In this study, risk factors are considered conditions or variables associated with higher likelihood of suicide or suicidal behavior based on preexisting evidence, and protective factors are those with adverse effect. Informed by previous intervention studies indicating gender differences in potentially effective programs to reduce suicide risk among the elderly, it was hypothesized that the protective role of resilience against suicidal behavior would be stronger for older men as compared to older women.

## Materials and Methods

### Participants and Procedure

Participants were recruited from Chuncheon, an urban-rural mixed community in Korea. The target number of sample was approximately 2000 older adults in the community, and the sample size was predetermined using a stratified cluster sampling procedure with a proportionate quota sampling strategy. Population sizes by gender and five age groups (65–69, 70–74, 75–79, 80–84, and ≥85 years old) were obtained from 25 administrative districts in Chuncheon. The sample sizes for higher and lower level administrative districts were determined based on the population size of gender by age groups. Eligible participants were 65 years and older, and could speak and understand Korean. Older adults residing in institutions such as nursing homes or hospitals were excluded from the study. A total of 4864 older adults were approached in the community, and 2034 among them agreed to participate in and completed the study. Response rate was 41.8%. The final sample included 833 men (41.0%) and 1201 women (59.0%).

A community-based survey was conducted via home visit by trained interviewers. All interviewers had at least 3 years of community survey experience and more than high school diploma. The mean survey experience of these interviewers was 5.04 years with the range of 3–19. All research personnel were trained by a doctoral level clinical psychologist regarding research ethics, interview procedure, and how to conduct suicide-risk assessment using the SBQ-R (see Materials for more information about the measure). Older adults who scored more than 7 on the SBQ-R were referred to community mental health centers upon agreement. Explanations about the aim of the study and contents of informed consent were presented verbally. All people could freely choose not to participate in the study without any disadvantages. For those who agreed to participate, all questions were read by research assistants. All participants provided written informed consent and received a small gift (less than $5 value) for the study participation. The study protocol was approved by Hallym University Institutional Review Board (IRB).

### Materials

The Suicidal Behaviors Questionnaire-Revised (SBQ-R; Osman et al., 2001) was developed to be used as a measure of suicidal behavior in clinical and non-clinical populations. The SBQ-R consisted of four items: (1) lifetime experience of suicidal thoughts and attempts (1 = never to 4 = I have attempted to kill myself, and really hoped to die); (2) frequency of suicidal ideation during the past 12 months (1 = never to 5 = very often, 5 or more times); (3) threat of suicide attempt (1 = no/never to 3 = yes/more than once, and really wanted to do it); and (4) self-reported likelihood of making suicidal behavior in the future (0 = never to 6 = very likely). The SBQ-R was translated into Korean and cross-checked by two doctoral-level psychologists and then confirmed by a bilingual person who is not in the field of psychology or psychiatry. The total scores of the SBQ-R range from 3 to 18, with the higher scores indicating a greater suicide risk. The cut-off score of 7 was suggested in classifying a high-risk group among non-clinical samples (Osman et al., 2001). The internal consistency coefficients of the SBQ-R were 0.76–0.88 in the Osman et al. (2001) study and 0.81 in this study.

The CD-RISC (Connor and Davidson, 2003) is a 25-item self-report measure that assesses one’s ability to cope with stressful life events in multiple domains of life. The CD-RISC measures a variety of characteristics of one’s resilience, such as the ability to control, goal-oriented active coping, adaptability to change, ability to tolerate negative affect, self-efficacy, personal competence, relationship security, and spirituality. The total scores of the CD-RISC range from 0 to 100 with the higher scores representing greater resilience. The participants rated each item on a five point Likert scale from 0 (not true at all) to 4 (true nearly all of the time). In this study, the Korean version of the CD-RISC (K-CD-RISC, Baek et al., 2010) was used. The internal consistency of the K-CD-RISC was 0.93 in Baek et al. (2010)’s study, and 0.95 in this study.

The Center for Epidemiologic Studies Depression Scale (CES-D; Radloff, 1977), a 20-item self-report questionnaire, measures depressive symptom severity in the last week, with a 4-point Likert scale (0 = rarely or none of the time, less than 1 day; 3 = most or all of the time, 5–7 days). In this study, a 10-item brief version of the CES-D (Kohout et al., 1993), translated into Korean by Shin (2011) was used. The brief version of the CES-D had good reliability among the elderly (Andresen et al., 1994; O’Halloran et al., 2014). The total scores of the brief CES-D range from 0 to 30, with the higher scores indicating greater depressive symptoms. The internal consistency of the 10-item brief version of the CES-D was 0.79 in Shin (2011)’s study and 0.87 in this study.

History of physical illnesses was assessed using the question of “Have you ever been diagnosed with any of the following illnesses by medical doctors?” Participants were responded to the question for 16 major physical illnesses such as hypertension, heart disease, stroke, cancer, or diabetes. The same question was used to assess history of depression. Also, sociodemographic information of age, gender, years of education, family income, and living status (living alone versus living together with family or others) was collected.

### Statistical Analysis

Descriptive statistics and gender comparisons for all variables were examined using t or χ^2^ tests. Correlation coefficients of the SBQ-R and all variables were examined, and Fisher’s *r* to z-transformation was used to examine gender differences. The Holm–Bonferroni method was used to adjust *p*-values for multiple comparisons. Next, hierarchical multiple regression was conducted to examine gender difference in the protective role of resilience for suicidal behavior. After controlling for sociodemographic covariates, physical illness, depressive symptoms, and depression history in the first step, the main effects of resilience and gender were examined in the second step, and then the interaction of gender by resilience were inserted in the third step. Finally, *post hoc* tests were conducted to depict the interaction effect found in the hierarchical multiple regression analyses (Aiken and West, 1991; Holmbeck, 2002).

## Results

### Preliminary Analysis

The mean age of the respondents was 74.50 (*SD* = 6.36) years old with a range of 65–98, and the average years of education was 6.12 (*SD* = 4.82). Of the sample, 53.8% were married or living with a partner, 41.9% were widowed, 2.6% were divorced, 1.4% were separated, and 0.2% were never married. The majority reported living with family (*n* = 1414, 69.5%) and 30.5% (*n* = 620) reported living alone.

As seen in **Table 1**, women were older, *t* = -5.66, *p* < 0.001, and less educated, *t* = 21.39, *p* < 0.001, compared to men. Also, women reported significantly higher scores on the CES-D (*M* = 4.90, *SD* = 4.67) than men (*M* = 3.90, *SD* = 4.62), *t* = -4.78, *p* < 0.001, indicating that women were more depressed than men. A significant gender difference was found in living status, in which a greater percentage of women reported living alone (42.1%) compared to men (13.7%), χ^2^ (1) = 187.82, *p* < 0.001. No significant gender difference was found in the SBQ-R, *t* = 1.16, *p* = 0.248.

**Table 1:** Preliminary analysis: A gender comparison of variables.

Variables	Total (*N* = 2034)	Men (*n* = 833)	Women (*n* = 1201)	Test	*p*
	*M* (*SD*)	*M* (*SD*)	*M* (*SD*)		
Age	74.50 (6.36)	73.56 (6.05)	75.15 (6.50)	*t* = -5.66	<0.001^†^
Years of education	6.12 (4.82)	8.67 (4.79)	4.36 (3.98)	*t* = 21.39	<0.001^†^
Family income^a^	3.11 (1.93)	3.49 (1.94)	2.84 (1.87)	*t* = 7.49	<0.001^†^
Living alone, *n* (%)	620 (30.5)	114 (13.7)	506 (42.1)	χ^2^ (*1*) = 187.82	<0.001^†^
Medical illness	2.02 (1.49)	1.61 (1.29)	2.30 (1.55)	*t* = -10.91	<0.001^†^
CES-D	4.49 (4.67)	3.90 (4.62)	4.90 (4.67)	*t* = -4.78	<0.001^†^
Depression history, *n* (%)	59 (2.9)	12 (1.4)	47 (3.9)	χ^2^(1) = 10.60	0.001^†^
CD-RISC	50.26 (15.87)	54.66 (16.56)	47.21 (14.62)	*t* = 10.46	<0.001^†^
SBQ-R	3.85 (1.99)	3.91 (2.03)	3.81 (1.97)	*t* = 1.16	0.248

*CES-D, Center for Epidemiologic Studies Depression Scale; CD-RISC, Connor-Davidson Resilience Scale; SBQ-R, Suicidal Behaviors Questionnaire Revised (SBQ-R); ^a^Family income: 1 = less than 500,000 KW (Approximately $428 USD), 2 = 500,000 to 990,000 KW (Approximately $428 to $848 USD), 3 = 1,000,000 to 1,490,000 KW (Approximately $857 to $1,277 USD), 4 = 1,500,000 to 1,990,000 KW (Approximately $1,285 to $1,705 USD), 5 = 2,000,000 to 2,450,000 KW (Approximately $1,714 to $2,099 USD), 6 = 2,500,000 to 2,990,000 KW (Approximately $2,142 to $2,562 USD), 7 = 3,000,000 KW (Approximately $2,571 USD) or above.*

*^†^With Holm–Bonferroni correction, a *p*-value lower than 0.025 is needed to be considered significant.*

The possible score range of the CD-RISC is 0 to 100. In the current sample, the mean score of the CD-RISC was 50.26 (*SD* = 15.87) and the score distribution was close to normal distribution (skewness index = -0.08, *SE*_skewness_ = 0.05; kurtosis index = 0.12, *SE*_kurtosis_ = 0.11). There was a significant gender difference in scores of the CD-RISC, in which men (*M* = 54.66, *SD* = 16.56) reported higher scores than women (*M* = 47.21, *SD* = 14.62), *t* = 10.46, *p* < 0.001.

Correlation coefficients of the SBQ-R and all variables were produced by gender, and Fisher’s *r* to z-transformation was used to examine whether these correlations differ by gender (see **Table 2**). With Holm–Bonferroni correction, the correlation coefficients between the SBQ-R and all variables were significant for both genders except age and years of education. A significant gender difference was found for the strength of correlations between the SBQ-R and the CD-RISC, *z* = -2.88, *p* = 0.004, in which the correlation between the SBQ-R and CD-RISC was stronger for older men, *r* = -0.32, *p* < 0.001, than women, *r* = -0.20, *p* < 0.001. On the contrary, a significant gender difference was found for the correlation between the SBQ-R and depression history, *z* = -3.55, *p* < 0.001. The correlation between the SBQ-R and depression history was stronger for older women, *r* = 0.33, *p* < 0.001, than men, *r* = 0.18, *p* < 0.001.

**Table 2:** Gender differences in correlation coefficients of the SBQ-R and variables.

	Men (*n* = 833)		Women (*n* = 1201)		
Variables	*r*	*p*	*r*	*p*	Fisher’s *z*	*p*
Age	0.02	0.640	-0.03	0.242	1.11	0.267
Years of education	-0.08	0.018	-0.07	0.014	-0.25	0.803
Family income^a^	-0.22	<0.001^†^	0.14	<0.001^†^	-1.66	0.097
Living alone^b^	0.19	<0.001^†^	0.08	0.005^†^	2.39	0.017
Physical illness	0.17	<0.001^†^	0.20	<0.001^†^	-0.68	0.497
CES-D	0.47	<0.001^†^	0.40	<0.001^†^	2.02	0.043
Depression history ^c^	0.18	<0.001^†^	0.33	<0.001^†^	-3.55	<0.001^††^
CD-RISC	-0.32	<0.001^†^	-0.20	<0.001^†^	-2.88	0.004^††^

*SBQ-R, Suicidal Behaviors Questionnaire Revised (SBQ-R); CES-D, Center for Epidemiologic Studies Depression Scale; CD-RISC, Connor-Davidson Resilience Scale; ^a^Family income was coded as 1 = less than 500,000 KW (Approximately $428 USD), 2 = 500,000 to 990,000 KW (Approximately $428 to $848 USD), 3 = 1,000,000 to 1,490,000 KW (Approximately $857 to $1,277 USD), 4 = 1,500,000 to 1,990,000 KW (Approximately $1,285 to $1,705 USD), 5 = 2,000,000 to 2,450,000 KW (Approximately $1,714 to $2,099 USD), 6 = 2,500,000 to 2,990,000 KW (Approximately $2,142 to $2,562 USD), 7 = 3,000,000 KW (Approximately $2,571 USD) or above; ^b^Living alone was coded as 0 = living with family or others, 1 = living alone; ^c^Depression history was coded as 0 = the absence of depression history, 1 = the presence of depression history.*

*^†^With Holm–Bonferroni correction, a *p*-value lower than 0.01 is needed to be considered significant.*

*^††^With Holm–Bonferroni correction, a *p*-value lower than 0.007 is needed to be considered significant.*

### Gender Difference in the Protective Role of Resilience against Suicidal Behavior

**FIGURE 1:**
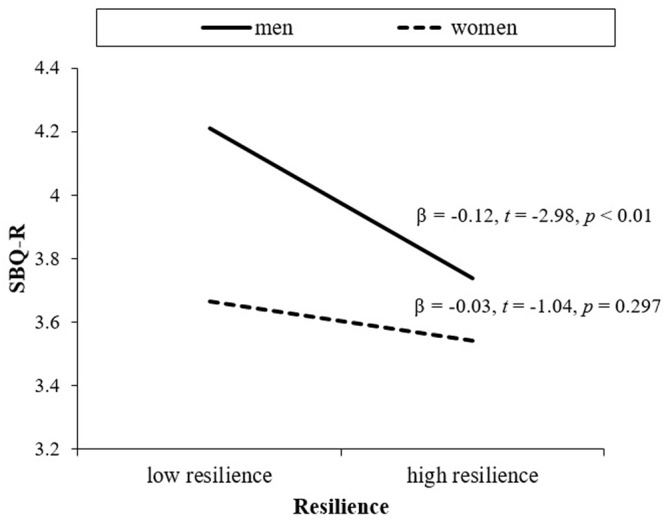
**Resilience and suicidal behavior in older men and women**.

Hierarchical multiple regression was conducted to examine the gender difference in the protective role of resilience against suicidal behavior among older adults. As seen in **Table 3**, after controlling for age, years of education, family income, living alone, physical illness, the CES-D, and depression history, the main effects of the CD-RISC, β = -0.01, *t* = -3.15, *p* = 0.002, and gender on the SBQ-R, β = -0.42, *t* = -4.44, *p* < 0.001 were significant. Specifically, lower resilience and being men increased scores of the SBQ-R. In an examination of the interaction effect of gender and the CD-RISC in the third step, the interaction was statistically significant, β = 0.01, *t* = 2.28, *p* = 0.023, indicating a gender difference. *Post hoc* tests indicated that the inverse relationship between the CD-RISC and the SBQ-R was significant only for men, β = -0.12, *t* = -2.98, *p* < 0.01, whereas the association was not statistically significant for women, β = -0.03, *t* = -1.04, *p* = 0.297 (see **Figure 1**).

**Table 3:** Hierarchical multiple regression: Gender difference of resilience on the SBQ-R.

Variables	β	*t*	*p*	Adjusted *R*^2^	Δ*R*^2^	*p*	*F*	*p*
Step 1				0.21	-	<0.001	77.83	<0.001
Age	-0.02	-3.04	0.002					
Years of education	0.01	1.38	0.167					
Family income	-0.09	-3.57	<0.001					
Living alone	-0.05	-0.56	0.574					
Physical illness	0.05	1.60	0.109					
CES-D	0.15	16.24	<0.001					
Depression history	2.05	8.53	<0.001					
Step 2				0.22	0.01	<0.001	64.47	<0.001
Gender^a^	-0.42	-4.44	<0.001					
CD-RISC	-0.01	-3.15	0.002					
Step 3				0.22	0.00	0.023	58.66	<0.001
Gender X CD-RISC	0.01	2.28	0.023					

*SBQ-R, Suicidal Behaviors Questionnaire Revised (SBQ-R); CES-D, Center for Epidemiologic Studies Depression Scale; CD-RISC, Connor-Davidson Resilience Scale; ^a^Gender was coded as 0 for men and 1 for women.*

## Discussion

The present study examined gender difference in the protective role of resilience against suicidal behavior in a community sample of older adults in Korea. Particularly, this study examined whether resilience would protect against suicidal behavior above and beyond well-known risk factors in older adults, and further such protective function of resilience would differ by gender. The results of the study indicated that gender moderated the relationship between resilience and suicidal behavior, in which the relationship between low resilience and suicidal behavior was found only for older men, but not for older women, after controlling for common risk factors including depression, physical illness, and sociodemographic variables.

This gender difference may be because the CD-RISC (Connor and Davidson, 2003) mostly measures individual, personal strengths and resources, which could be more applicable to men than women. For older women, it is possible that relational resources are more related to their resilience against suicidal behavior. Considering previous research indicating unsuccessful effects of intervention on risk factors among older men (Lapierre et al., 2011), intervention promoting resilience may be an alternative option in reducing suicidal behavior for older men.

The findings of this study indicate the need for developing gender-specific preventive intervention strategies. Depression screening and follow-up care seem to be useful for suicide prevention, but as indicated in Lapierre et al.’s (2011) review, older men were less inclined to use mental health services and preferred action or problem-solving oriented programs. Hinton et al. (2006) reported that older men were rather reluctant to participate in mental health care due to traditional masculine value or stigma of mental illness. Our results indicate that strength-focused, competence-based preventive intervention to promote resilience or personal strengths could be a promising option for older men.

One important matter, then, is what to modify to improve resilience in older adults. To answer this question, we need to improve our understanding regarding the construct of resilience and what correlates with resilience in older adults. A few studies reported that resilience in older adults was associated with optimism, successful aging, depression, physical functioning, daily functioning, living with others, and days spent with family and friends per week (Hardy et al., 2004; Lamond et al., 2008; Jeste et al., 2013). Although further studies are needed, these variables are all potential ingredients in psychosocial intervention to improve resilience in older adults.

Limitations of the study should be noted. First, the older adults who participated in the study mostly lived in urban–rural mixed areas in South Korea and those living in nursing homes and hospitals were excluded for the study. Thus, further studies are needed for other samples, such as urban-residing or clinical samples. Second, we used a resilience measure covering a broad, heterogeneous concept of resilience. It is possible that older men and women have different domains of resilience associated with suicidal behavior, which we were unable to examine. Additionally, it was possible that men tended to exaggerate their resilience than women due to their masculine value. Third, most of the scales used for this study were self-report questionnaires that were not developed for oral administration. We did so because older adults vary in terms of reading capability due to various reasons such as vision or educational level. Further research to develop measures specific to older adults is needed. Finally, a single question was used to assess medical history about 16 major physical illnesses and depression. It should be considered that there was a possibility of underreporting.

Strengths of the study included a relatively large community sample of older adults, and the use of the multistage cluster sampling strategies to obtain a representative sample in consideration of regional differences in age and gender distribution. Considering the lack of studies examining gender differences in suicidal behavior in the elderly population, this study provides preliminary data indicating gender difference in factors related to suicidal behavior.

## Conclusion

This study highlights gender differences in resilience against suicidal behavior above and beyond the effects of common risk factors in the elderly. Particularly, the results of the study indicate the protective role of resilience for older men. Considering that suicide rate of older men is very high and their participation rates in prevention programs focusing on mental health or social activities are low, alternative prevention efforts are needed for this population. The present study indicates that community-based, universal approach focusing on enhancement of resilience could be an alternative approach for older men. Differential intervention strategies for men and women need to be developed to implement effective suicide prevention programs. For older men, intervention programs focusing on personal strengths or assets, coping skills, or resilience would help reduce suicide risk.

## Author Contributions

Both authors have made substantial contributions to the conception and design of the study, data analysis and interpretation of the data. SY drafted the manuscript. Both authors contributed to critical revisions, and approved the final version to be published.

## Conflict of Interest Statement

The authors declare that the research was conducted in the absence of any commercial or financial relationships that could be construed as a potential conflict of interest.
